# Weil's disease: rapid recovery following corticosteroid treatment: a case report

**DOI:** 10.1590/S1678-9946202668035

**Published:** 2026-05-15

**Authors:** Ahmet Melih Şahin, İrem Atçı-Koç, Sinan Çetin

**Affiliations:** 1 Giresun University, Medical Faculty, Department of Infectious Diseases and Clinical Microbiology, Giresun, Turkey

**Keywords:** Leptospirosis, Weil's disease, Corticosteroid therapy, Hyperbilirubinemia, Thrombocytopenia

## Abstract

Leptospirosis is a zoonotic infection with a range of clinical findings, ranging from a mild, self-limited illness to a severe, life-threatening disease known as Weil's disease. The role of adjunctive corticosteroid therapy in severe leptospirosis remains controversial. We report a 41-year-old male with a history of intravenous drug use, regular alcohol consumption, and exposure to contaminated freshwater, who presented with fever, myalgia, vomiting, jaundice, thrombocytopenia, and acute kidney injury. Serological testing via the microagglutination test (MAT) was negative at admission, and empirical antibiotic therapy with ceftriaxone was initiated. Despite appropriate antimicrobial treatment and supportive care, including hemodialysis, the patient developed progressive hyperbilirubinemia and persistent thrombocytopenia. A repeat MAT performed one week later confirmed infection with *Leptospira interrogans* serovar Pomona. Due to ongoing clinical deterioration, methylprednisolone (1 mg/kg/day) was initiated, resulting in a dramatic clinical and laboratory improvement within 24 h. This rapid response suggests a potential role for corticosteroid therapy in selected patients with severe leptospirosis; however, further studies are required to establish its efficacy and safety.

## INTRODUCTION

Leptospirosis is a zoonotic disease caused by pathogenic spirochetes of the genus *Leptospira*. Infection occurs via direct or indirect contact with the urine or tissues of infected animals. The estimated annual number of cases worldwide exceeds one million, with approximately 59,000 deaths reported each year^1^.

Although cattle, pigs, horses, and dogs may also serve as reservoirs, rodents play the most significant role in transmitting the disease to humans. Transmission typically occurs through contact with water or soil contaminated with the urine of infected animals. Spirochetes usually enter the human body through mucous membranes or broken skin^2,3^.

Incubation period averages 5–14 days, with cases of up to 30 days having been reported. The disease may initially present nonspecific symptoms, and most patients have a favorable prognosis. However, a severe clinical course is observed in approximately 10% of cases which may develop jaundice, arrhythmias, bleeding, hemodynamic instability, liver failure, pulmonary failure, renal failure, and aseptic meningitis. The case fatality rate among patients with severe disease ranges from 5% to 15%^1,4^.

The standard serological method used for diagnosis is the microagglutination test (MAT). Low serological sensitivity during the early phase of the disease may lead to delays in initiating antibiotic therapy. In such patients, other treatment options may be required to prevent clinical deterioration despite early diagnosis and effective antimicrobial therapy^1,5^. For this purpose, plasmapheresis and corticosteroid therapy have also been used in selected cases^6,7^.

The clinical course of leptospirosis is generally described as consisting of two phases: an acute phase and an immune phase. During the acute phase, clinical manifestations are mainly associated with bacterial dissemination and direct tissue invasion. In severe forms of the disease, however, the host immune response plays a more prominent role. During the immune phase, an exaggerated proinflammatory cytokine response may contribute to endothelial dysfunction. Although the exact pathophysiology has not been fully elucidated, this mechanism is thought to play an important role in the development of severe organ involvement, including pulmonary hemorrhage, acute kidney injury, and hepatic dysfunction. The thrombocytopenia and hyperbilirubinemia observed in severe leptospirosis are also believed to be associated with endothelial damage and inflammatory processes^8,9^. Corticosteroids have been proposed to suppress the excessive inflammatory response, thereby reducing endothelial damage and capillary leakage, and potentially limiting the progression of organ dysfunction. However, the available evidence remains limited, and there is currently no clear consensus regarding the efficacy and safety of corticosteroid therapy^2,3,10^.

Here, we present a patient who was MAT-negative in the early stage, showed no clinical improvement despite standard treatment, and had persistently elevated bilirubin levels and ongoing thrombocytopenia, but who responded rapidly to corticosteroid therapy.

### Ethics

Ethical approval was not required for this case report according to national regulations; therefore, it was not submitted to an ethics committee. Written informed consent was obtained from the patient.

## CASE REPORT

A 41-year-old male patient presented to the emergency department with complaints of diarrhea, vomiting, myalgia, and jaundice that had started four days earlier. His medical history revealed that he lived in the city center, worked as a carpenter, had been fishing in a stream one week earlier, and had consumed wild mushrooms two days prior to admission. He reported that his symptoms had begun approximately one week earlier and had progressively worsened, with the development of jaundice and decreased urine output over the last two days. He had no known chronic medical conditions but had a history of intravenous drug use and regular alcohol consumption.

On physical examination, his blood pressure was 110/60 mmHg, pulse rate was 130 beats/min, and body temperature was 37.8 °C. The patient's general condition was stable. The skin and sclerae were icteric. Apart from right upper quadrant tenderness, no abnormal findings were detected on physical examination. There were no signs of meningeal irritation or focal neurological deficits.

Laboratory evaluation revealed a hemoglobin level of 14.2 g/dL, white blood cell count of 12,300/μL (91% neutrophils), platelet count of 37,000/μL, total bilirubin of 11.32 mg/dL, conjugated bilirubin of 10.2 mg/dL, alanine aminotransferase (ALT) of 44 U/L, aspartate aminotransferase (AST) of 71 U/L, urea of 153 mg/dL, creatinine of 5.5 mg/dL, and C-reactive protein of 327 mg/L. Venous blood gas analysis showed a pH of 7.34 and a bicarbonate level of 20 mmol/L.

Hepatobiliary ultrasonography revealed millimetric echogenicities in the liver and increased gallbladder volume with biliary sludge. Magnetic resonance cholangiopancreatography demonstrated findings suggestive of acute or toxic hepatitis; the intrahepatic bile ducts and common bile duct were normal, with no evidence of acute cholecystitis. Perirenal changes were consistent with acute kidney injury (AKI).

The patient was admitted with preliminary diagnoses of fungal intoxication, acute viral hepatitis, cholecystitis, hantavirus infection, and leptospirosis. Blood cultures were obtained, and ceftriaxone (2 g/day intravenously) therapy was initiated along with supportive treatment. Serum samples sent for MAT and immunofluorescent antibody testing for leptospirosis and hantavirus infection, respectively, were initially negative. Serological tests for HAV IgM, HBsAg, anti-HBc IgM, anti-HCV, anti-HIV, anti-HEV IgM, CMV IgM, EBV VCA IgM, and HSV IgM were all negative.

Fungal intoxication was excluded based on progressive renal dysfunction, increasing bilirubin levels, and the normalization of initially elevated transaminase levels; additionally, the same mushrooms were consumed by other family members who remained asymptomatic. Toxicological screening for amphetamines, benzodiazepines, cocaine, opiates, and tetrahydrocannabinol was negative. Due to anuria, the patient was initiated on hemodialysis.

Total bilirubin levels increased to 15.2 mg/dL, and platelet counts decreased to 15,000/μL. A second MAT performed seven days later detected antibodies against *Leptospira interrogans* serovar Pomona at a titer of 800. Ceftriaxone therapy was completed for seven days, and platelet transfusions were administered. On day 15, total bilirubin reached 23 mg/dL, while platelet counts remained low at 37,000 /μL despite platelet transfusions.

**Figure 1:**
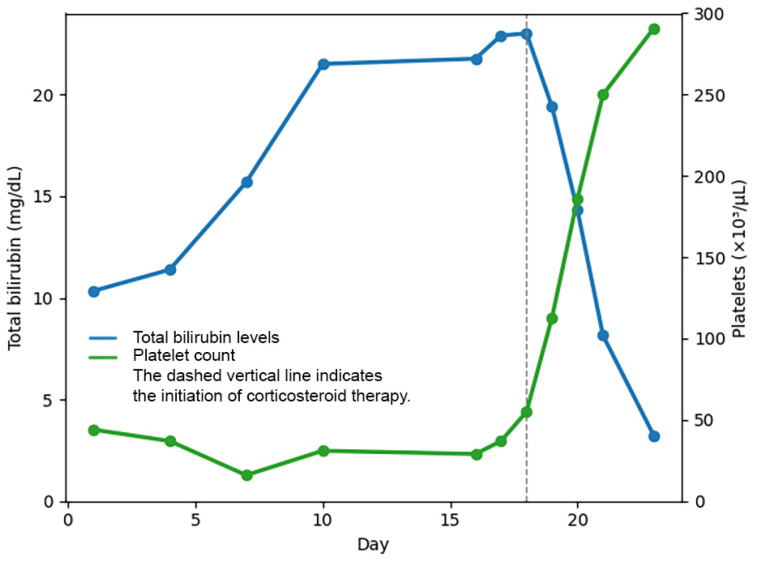
Temporal changes in total bilirubin levels and platelet count during hospitalization.

As urine output gradually resumed, the patient underwent three sessions of hemodialysis. On day 18, due to persistent fatigue and nausea, intravenous methylprednisolone at a dose of 1 mg/kg/day was initiated when the total bilirubin was 23.01 mg/dL and the platelet count was 55,000/μL. A dramatic clinical and laboratory response was observed within 24 h, with total bilirubin decreasing to 19.05 mg/dL and platelet count increasing to 113,000 /μL (Figure 1).

**Figure 2:**
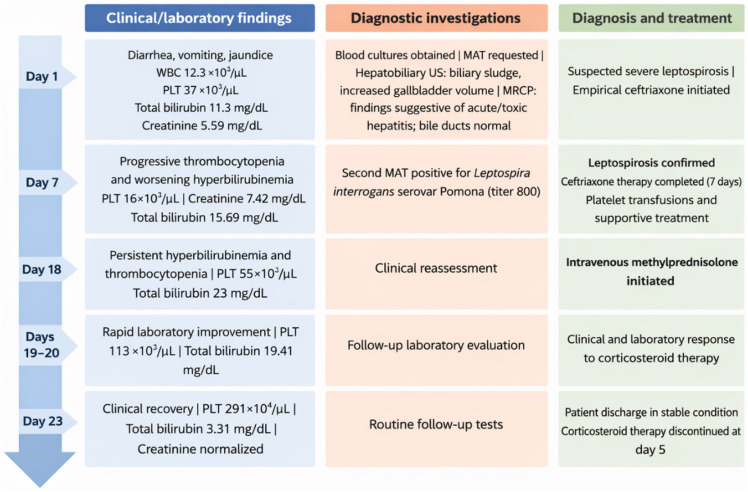
Timeline of the case.

Subsequently, the patient's clinical condition and laboratory parameters improved rapidly. Urine output increased progressively, entered a polyuric phase, and later normalized. After five days of corticosteroid therapy, total bilirubin decreased to 3.21 mg/dL and platelet count increased to 291,100/μL. Corticosteroid therapy was discontinued after 5 days without tapering. The patient was discharged on day 23 with improved renal function, resolution of symptoms, and no fever. At outpatient follow-up on day 29, complete clinical recovery and normalization of laboratory parameters were observed (Table 1 and Figure 2).

**Table 1:** Laboratory parameters during hospitalization.

Parameter	Day 1	Day 4	Day 7	Day 10	Day 16	Day 17	Day 18^a^	Day 19	Day 20	Day 21	Day 23
WBC (×10³/µL)	16.23	12.30	13.02	12.04	13.75	18.03	12.87	10.30	7.95	7.76	12.54
Platelets (×10³/µL)	44	37	16	31	29	37	55	113	186	250	291
Creatinine (mg/dL)	4.69	5.50	7.42	6.57	4.90	3.31	2.13	1.46	1.06	0.85	0.74
Potassium (mmol/L)	3.8	3.6	3.9	4.2	3.7	3.9	3.6	4.0	3.8	4.2	3.9
Total bilirubin (mg/dL)	10.34	11.39	15.69	21.51	21.77	22.91	23	19.41	14.31	8.15	3.21
Conjugated bilirubin (mg/dL)	8.74	10.34	14.06	18.38	20.46	19.35	19.21	16.78	11.24	7.10	3.01
ALT (U/L)	43	44	34	28	32	32	32	28	32	58	89

a Methylprednisolone was initiated.

## DISCUSSION

Although leptospirosis is generally known to have a favorable course, it should not be overlooked as it can lead to severe complications and death. Failure to recognize the disease in its early stages or include it in the differential diagnosis negatively affects prognosis. In patients with suspected leptospirosis, starting antimicrobial treatment as soon as possible, without waiting for the results of diagnostic tests, is effective in reducing the severity and duration of the infection^1^.

Mild cases usually present with non-specific flu-like symptoms and can easily be confused with a viral infection, while more severe cases may present with arrhythmia, bleeding, hemodynamic disorders, liver failure, renal failure, pulmonary hemorrhage, meningitis, or sepsis. Accordingly, many diseases must be considered in the differential diagnosis^4,11^. In our case, which presented with diarrhea, vomiting, joint pain, and jaundice, viral serological tests for anti-HAV IgM, HBsAg, anti-HBc IgM, anti-HCV, anti-HIV, anti-HEV IgM, anti-CMV IgM, anti-EBV IgM, anti-HSV IgM, and Hantavirus IFA IgM and IgG were negative. The MAT test requested for leptospirosis was also initially negative in the patient, whose renal function rapidly deteriorated while thrombocytopenia worsened and jaundice increased. The patient's occupation as a carpenter and his recent history of entering a river suggested that the early leptospirosis MAT result could be a false negative. The sensitivity of serology is highest in the second week of the disease^1^. At this stage, a second MAT test yielding a positive result is important for confirming a false-negative initial result in the presence of strong history and clinical suspicion^2^. Conversely, it should be borne in mind that false positives may also occur due to cross-reactivity of antibodies in infections such as syphilis, Lyme disease, typhoid fever, or dengue fever^12^.

Although the mechanism of jaundice development in leptospirosis is not fully understood, it is thought that leptospires attack hepatocytes in the liver, causing damage that leads to bile leakage from the bile ducts into the sinusoidal blood vessels, resulting in high conjugated bilirubin levels^13^. A review of the literature reveals at least one case of leptospirosis with a total bilirubin level reaching 74.3 mg/dL^14^.

In our case, methylprednisolone treatment was initiated due to persistent hyperbilirubinemia and thrombocytopenia, and a dramatic clinical and laboratory response was achieved within a short period. Although corticosteroid treatment is not commonly used in leptospirosis patients, a small number of conflicting results are noted in the literature. Two cases reported from Turkey and Malaysia emphasized rapid clinical and laboratory parameter improvement with corticosteroid therapy^5,15^. A study evaluating 149 leptospirosis cases in Sri Lanka reported that early methylprednisolone therapy had a significant effect on mortality. A review examining five studies, four of which involved severe leptospirosis cases, showed that early-stage methylprednisolone treatment was effective in patients with pulmonary involvement, while one study indicated that it was ineffective and could increase the risk of nosocomial infection^16^. Similarly, in a review by Petakh *et al*.^10^, it was stated that further studies are needed on the benefit and efficacy of corticosteroid treatment in patients with severe leptospirosis. Moreover, a meta-analysis evaluating studies from four different countries found no clear evidence regarding the efficacy of corticosteroid treatment^3^.

Leptospirosis has an acute and an immune phase. Due to renal failure, jaundice, hemodynamic instability, severe thrombocytopenia, and a prolonged clinical course, our case represents a severe form of leptospirosis. The rapid improvement in bilirubin levels and normalization of thrombocytopenia following methylprednisolone administration may be related to the mechanism whereby corticosteroids suppress the immune response, thereby reducing organ damage. The unclear immunological mechanism prevents the clear identification of interactions between *Leptospira* and the host immune system. Consequently, the few reviews conducted also indicate that the mechanisms by which corticosteroids prevent disease progression remain unclear^2,3,10^.

## CONCLUSION

Our case demonstrates that the microagglutination test may be negative in the early stages of leptospirosis and that a detailed history and strong clinical suspicion are decisive in establishing the diagnosis. Moreover, the persistence of hyperbilirubinemia and thrombocytopenia despite standard antibiotic and supportive treatment suggests that immune-mediated mechanisms may play a role in the pathogenesis of the disease. The rapid clinical and laboratory response observed with corticosteroid treatment supports the potential therapeutic benefit of corticosteroids in selected severe leptospirosis cases; however, controlled clinical trials are needed to clarify the efficacy and safety of this approach.

## Data Availability

The complete anonymized dataset supporting the findings of this study is included within the article itself.
